# Higher mobility of butterflies than moths connected to habitat suitability and body size in a release experiment

**DOI:** 10.1002/ece3.1187

**Published:** 2014-09-12

**Authors:** Mikko Kuussaari, Matias Saarinen, Eeva-Liisa Korpela, Juha Pöyry, Terho Hyvönen

**Affiliations:** 1 Finnish Environment Institute, Natural Environment CentreP.O. Box 140, FI-00251, Helsinki, Finland; 2 Castréninkatu 10 b 17FI-00530, Helsinki, Finland; 3 MTT Agrifood Research Finland, Plant Production ResearchFI-31600, Jokioinen, Finland

**Keywords:** Animal movement, dispersal propensity, experimental study on migration, habitat preference, interspecific differences in mobility, mark-release-recapture study, release habitat suitability, species traits, variation in dispersal ability, wingspan

## Abstract

Mobility is a key factor determining lepidopteran species responses to environmental change. However, direct multispecies comparisons of mobility are rare and empirical comparisons between butterflies and moths have not been previously conducted. Here, we compared mobility between butterflies and diurnal moths and studied species traits affecting butterfly mobility. We experimentally marked and released 2011 butterfly and 2367 moth individuals belonging to 32 and 28 species, respectively, in a 25 m × 25 m release area within an 11-ha, 8-year-old set-aside field. Distance moved and emigration rate from the release habitat were recorded by species. The release experiment produced directly comparable mobility data in 18 butterfly and 9 moth species with almost 500 individuals recaptured. Butterflies were found more mobile than geometroid moths in terms of both distance moved (mean 315 m vs. 63 m, respectively) and emigration rate (mean 54% vs. 17%, respectively). Release habitat suitability had a strong effect on emigration rate and distance moved, because butterflies tended to leave the set-aside, if it was not suitable for breeding. In addition, emigration rate and distance moved increased significantly with increasing body size. When phylogenetic relatedness among species was included in the analyses, the significant effect of body size disappeared, but habitat suitability remained significant for distance moved. The higher mobility of butterflies than geometroid moths can largely be explained by morphological differences, as butterflies are more robust fliers. The important role of release habitat suitability in butterfly mobility was expected, but seems not to have been empirically documented before. The observed positive correlation between butterfly size and mobility is in agreement with our previous findings on butterfly colonization speed in a long-term set-aside experiment and recent meta-analyses on butterfly mobility.

## Introduction

Dispersal ability is a key factor affecting occurrence patterns and population trends in animals (Ewers and Didham 2006). Ongoing changes in land use and climate also pose strong selective pressures on species traits that are connected to animal mobility (Bonte et al. 2012; Baguette et al. 2013). An increased need to understand the impacts of environmental change at population and community levels has recently attracted much interest in the measurement of mobility differences across individuals, populations, and species (Bowler and Benton 2005; Clobert et al. 2012). However, despite the accumulating experience in estimating mobility (Nathan et al. 2008), producing reliable multispecies comparisons has remained a challenging task. Here, we used butterflies and moths for a multispecies mobility comparison to examine differences in dispersal ability among species and between species groups. Butterflies are one of the most popular groups in animal mobility research (Stevens et al. 2010), whereas knowledge on other insect groups, even among Lepidoptera, has remained scanty.

Several previous studies on butterflies have demonstrated the important role of interspecific mobility differences in species distributions and species responses to habitat and climate change. For example, the effects of habitat fragmentation have been shown to differ between butterfly species with varying mobility (Öckinger et al. 2009, 2010). Öckinger et al. (2010), using body size as a proxy for mobility, showed that butterfly species with low mobility have been most strongly affected by habitat loss and other studies have reported similar results. Kotiaho et al. (2005) found that threatened butterfly species are characterized by low mobility, and the meta-analysis by Thomas et al. (2011) showed that dispersal ability is one of the main drivers of long-term butterfly population trends. These results indicate that dispersal ability may crucially affect how species can cope with global threats such as climate change and habitat loss and fragmentation.

Moreover, recent studies have highlighted the importance of intraspecific variation in mobility and that relatively fast microevolutionary changes in dispersal ability and emigration propensity may play a significant role when species are adapting to changing environments (Merckx et al. 2003; Schtickzelle et al. 2006; Duplouy et al. 2013). Fast evolutionary changes may influence ecological population dynamics and vice versa, potentially causing complex eco-evolutionary dynamics in dispersal (Hanski and Mononen 2011). However, the large number of factors influencing evolution of dispersal complicates predictions on what would be the optimal dispersal strategy in different landscapes and in case of different population structures (Clobert et al. 2012).

Butterfly mobility has been empirically studied using a number of different approaches (Stevens et al. 2010; Sekar 2012). The most popular approach has been to conduct mark-release-recapture (MRR) studies in natural butterfly (meta)populations (Hovestadt and Nieminen 2009). However, mobility estimates from different single-species MRR studies are not directly comparable, because the results are strongly dependent on the spatial scale (Schneider 2003; Franzén and Nilsson 2007) and landscape structure (Mennechez et al. 2003; Dover and Settele 2009) of different studies. Manipulative experimental approaches have enabled to answer more specified questions concerning different components of butterfly mobility and to carry out intra- and interspecific comparisons. However, experimental releases of butterflies in the field (Söderström and Hedblom 2007; Kallioniemi et al. 2014) and studies conducted in large habitat cages (Norberg et al. 2002; Hanski et al. 2006) have been relatively restricted in spatial scale and have rarely involved more than two species.

Because of the great demand for comparable mobility estimates in community ecological studies, there is an obvious need for empirical studies producing comparable mobility estimates for a larger number of species simultaneously and in standardized conditions. We produced such estimates by experimentally releasing a large number of marked individuals of 60 butterfly and diurnal moth species in a large set-aside field and then collecting recaptures within the study landscape. Our aim was to collect a sufficient amount of comparable data in order to analyse interspecific differences in mobility and test our hypotheses on the effects of specific species traits on butterfly mobility based on earlier studies. More specifically, we aimed to answer the following study questions: (1) Do butterflies differ significantly from geometroid and noctuoid moths in mobility? (2) Does body size (wingspan) explain mobility differences between butterfly species? (3) Which other species traits affect mobility differences between butterfly species?

Based on previous studies on moths (Nieminen 1996; Nieminen et al. 1999), we hypothesized geometroids to be less mobile than noctuoids. Our expectation for the relationship between butterfly and moth mobility was less clear, because much variation has been reported in both species groups and direct multispecies comparisons between butterflies and moths have been lacking. However, our earlier results of a six-year set-aside experiment showed that butterflies colonized the set-aside faster than diurnal moths (Alanen et al. 2011), suggesting higher mobility in butterflies than moths.

Based on recent meta-analyses on butterfly mobility (Stevens et al. 2010, 2012; Sekar 2012) and our own results on colonization speed in butterflies (Alanen et al. 2011), we hypothesized mobility to increase with increasing body size (wingspan). The motivation to test the role of a set of other species traits stems from recent studies reporting significant effects of various traits on butterfly mobility (Stevens et al. 2010, 2012; Sekar 2012). Furthermore, we used the opportunity offered by our experimental set-up to test also the effect of release habitat suitability on mobility of species originating from different habitat types, hypothesizing that decreasing habitat suitability would increase emigration rate (Bowler and Benton 2005). Finally, we also considered the potential effects of phylogenetic relatedness on butterfly mobility. Characteristics of closely related species are often more similar compared with distantly related species, and thus the assumption of independent data points may be violated in comparative analyses including multiple species (Ives and Zhu 2006).

## Materials and Methods

### Experimental design and study area

**Figure 1:**
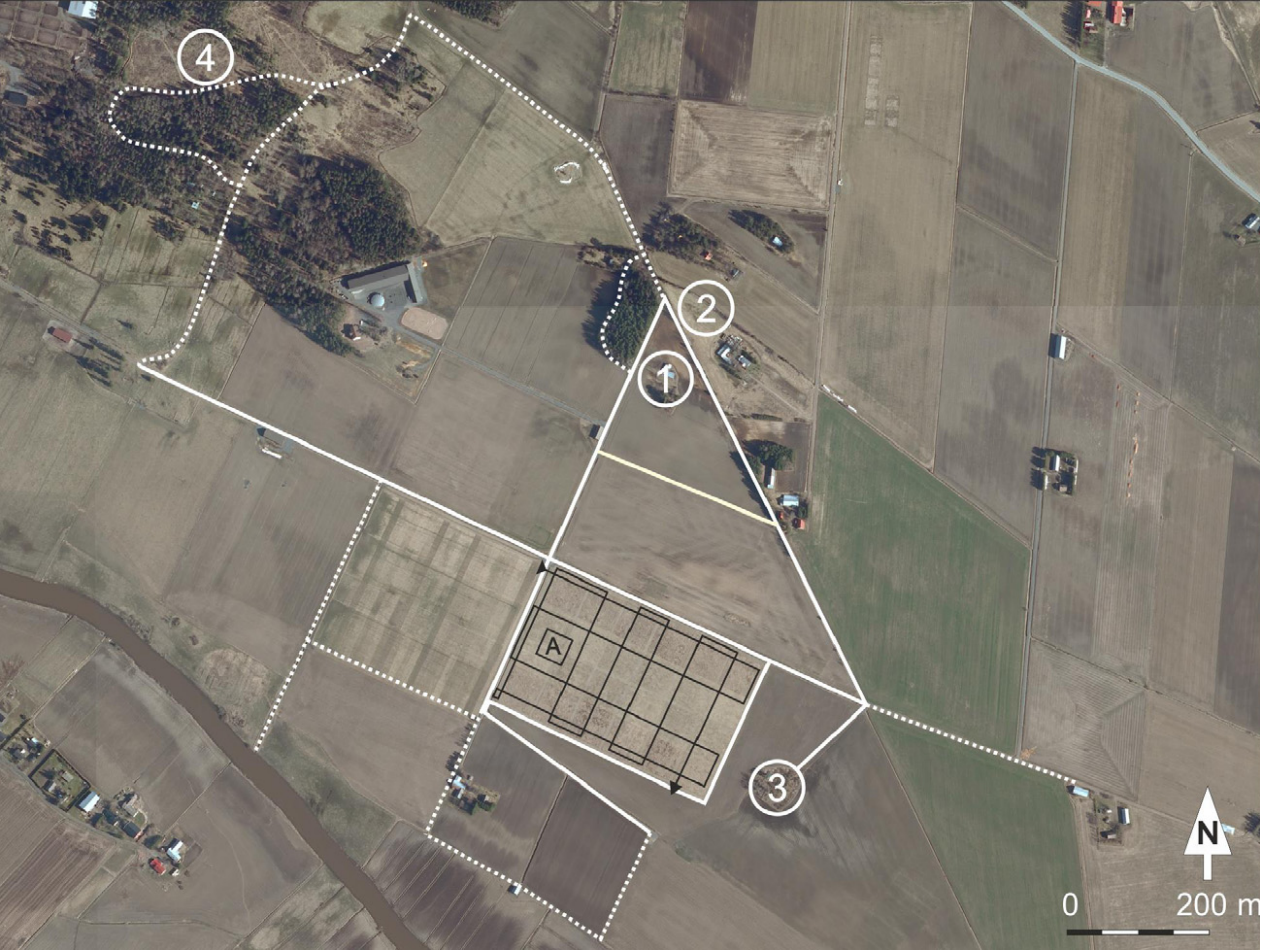
Aerial photograph of the study area. Letter A indicates the release area within the focal set-aside field. The black line with arrows indicates the 2500-m-long transect in which marked individuals were systematically searched. Solid white lines show the searching routes outside the release set-aside, and dashed white lines show the routes which were walked less frequently. Numbers 1–4 indicate favorable butterfly and moth habitats, which were used both for collecting individuals for the releases and for searching recaptures of emigrated individuals; especially sites 1 (abandoned farmyard and a sheltered forest edge) and 2 (semi-natural grassland patch) attracted many emigrants.

The experiment had a simple design in which marked lepidopteran individuals were released daily in a 25 m × 25 m release area within a 11-ha set-aside field, which was established eight years earlier (Fig.1; for the former six-year set-aside experiment, see Alanen et al. 2011). Movement distances of the marked individuals were then systematically recorded by recapturing them at different distances from the release area both within and outside the set-aside field (Fig.1). This design enabled us to record distance moved and emigration rate in a comparable manner for a larger set of butterfly and moth species than to our knowledge in any previous study.

The release set-aside field was located in Ypäjä, southwestern Finland (ETRS-TM35FIN N 6745551 E 299807), in an agricultural landscape dominated by spring cereal production. The landscape surrounding the set-aside field was flat and open agricultural land in all directions except toward the northwest, where there was a mosaic area of forests, species-rich semi-natural grasslands, and built-up areas starting from *c*. 600 m from the set-aside (Fig.1). The release set-aside was occupied by a relatively diverse community of grassland butterflies and diurnal moths, with many species even more abundant at the time of our release experiment than in year 2008, when the six-year set-aside experiment ended (see Table S1). For instance, *Lycaena hippothoe* had clearly established a local population on the set-aside after year 2008.

### Butterfly and moth releases

A total of 2011 butterfly and 2367 moth individuals belonging to 32 butterfly and 28 moth species were marked and released in the 25 m × 25 m release area within the set-aside field (for a detailed list of released species, see Table S1; nomenclature according to Kullberg et al. 2002). Individuals for the releases were collected from the set-aside field (40% of released individuals) as well as from the surrounding landscape (nine sites, 60% of individuals). The nine sites were located 50–3800 m from the release set-aside field and were good butterfly habitats, mostly patches of semi-natural grasslands and sheltered, sunny forest edges with some semi-natural vegetation. These sites were selected in order to maximize both the number of individuals and species released in the experiment. Collecting (unmarked) individuals from these sites for the releases also effectively served in collecting recaptures of marked individuals that had already emigrated from the release set-aside field (see below).

Butterflies and diurnal moths were marked, released, and recaptured daily during two study periods: from 30 May to 11 June and from 28 June to 14 July 2011. The first period covered the flight season of early summer species in southwestern Finland, whereas the second period covered the flight season of mid-summer species. This procedure enabled us to cover a large proportion of butterfly and diurnal moth species’ occurrence during the summer season. The weather was mostly warm and sunny (i.e., favorable for lepidopteran activity) during the two study periods.

Usually, individuals were collected for the releases from the release set-aside field during the morning and from the surrounding landscape during the afternoon. Butterflies were always marked with an individual number on the wing using a fine-point pen, whereas other lepidopteran species were marked with a color spot made on the wing with a thicker marker pen. The latter was performed by gently pressing the pen through the butterfly net without taking the moth individual in hand, in order to avoid damaging its fragile wings. Immediately after marking, each individual was placed individually within a 120-ml plastic container which was then stored in a cool box in order to keep the marked individuals inactive before the release.

Individuals marked within the release set-aside during the morning session were released close to the center of the 25 m × 25 m release area daily approximately at 12 o'clock, whereas the marked individuals collected from the surrounding landscape were typically released between 16 and 18 o'clock. In the release area, the butterflies and moths were gently placed individually on plant leaves and flowers. Recaptures were never collected within the 25 m × 25 m release area.

### Protocol for recaptures

In collecting data on movements of released butterflies and moths, the focus was on both within set-aside movements and movements to the surrounding landscape. Therefore, recaptures were searched daily in a systematic way at different distances from the release area, both in the release set-aside and in its surroundings.

Approximately one hour was spent on collecting recaptures within less than 100 meters from the release area every morning. Such a high effort was directed on the relatively close vicinity of the release area in order to ascertain at least some recaptures from as many released species as possible, including the least mobile species. In addition, the whole release set-aside field was systematically searched through by walking a 2500-m-long constant transect (Fig.1) every day. Approximately similar effort was directed on gathering recaptures of emigrated individuals in the surrounding landscape. Fig.1 shows the routes along field margins and road verges in the vicinity of the release set-aside field in which recaptures were searched for as often as time allowed (almost daily). In addition, four favorable butterfly and moth habitats (numbers 1–4 in Fig.1) turned out to attract many emigrated individuals, and therefore, these areas were visited almost daily. In summary, an area of *c*. 1 km^2^ was well-surveyed daily, whereas in total recaptures were collected from an area of *c*. 4 km^2^ in size.

For each recaptured individual, the following information was recorded: date, time, species, sex, individual number (for butterflies), and the exact location of the recapture, marked on an aerial photograph of the area.

### Measurement of movement parameters

Two main measures of mobility were recorded for each species with recaptures: average distance moved and emigration rate.

Distance moved was measured for each recaptured butterfly individual as the distance between the release point and the location of the last recapture, thereby each individual contributed to the results only once. For diurnal moths, which were not marked individually, the distance from the release point was recorded for every recapture point. Distances moved were measured from the aerial photographs in which the recapture points were marked in the field. For the statistical analyses, distances moved were ln-transformed after which they followed a normal distribution. An individual was considered as emigrated, if it was recaptured outside the release set-aside field. Based on the same logic as with distance moved, only the last recapture of a butterfly individual was used for indicating emigration, irrespective of its previous recapture records. In contrast, all recaptures of moths were considered as independent observations.

As a third measure potentially related to mobility, the proportion of recaptured individuals was recorded for all studied species. In previous studies on lepidopteran mobility, increasing fraction of disappeared (i.e., not recaptured) individuals has sometimes been considered as an indication of increasing mobility or emigration (Kuussaari et al. 1996; Merckx et al. 2009). In contrast to the other two mobility measures which are solely based on recaptures, all released lepidopteran individuals contributed to this measure and thus the fraction of recaptured individuals could potentially give some additional information on mobility.

In order to facilitate an unbiased comparison of mobility between butterflies and moths in statistical analyses, we also calculated all three mobility measures for butterflies using the same logic as in moths, that is, treating each butterfly recapture as a separate data point in the data set.

### Species traits

The analyses on the role of species traits focused only on butterflies as published species trait data are scanty for moths. The following six species traits were examined in order to explain observed mobility differences in butterflies: body size, adult habitat specificity and preference, larval host plant specificity and host plant type, and release habitat suitability. Body size was measured as a continuous variable, whereas all the other species traits were measured as categorical variables. The trait classifications for each studied species are shown in Table S2.

Body size of each species was measured as the average female wingspan (in mm), based on the Finnish butterfly handbook by Marttila et al. (1990). Adult habitat specificity was classified as a binary variable: habitat specialists occupying one or two and generalists occupying more than two habitat types following Ekroos et al. (2010) and originally based on Komonen et al. (2004). Habitat preference had three classes: forest edges and clearings, semi-natural grasslands, and field margins in open farmland, following Kuussaari et al. (2007). The specificity of larval host plant use was measured as a binary variable: mono- and oligophagous species feeding only on one host plant genus and polyphagous species feeding on more than one plant genus, based on Komonen et al. (2004). Larval host plant type was classified to the following four categories: woody plants (i.e., trees and shrubs as well as species in the family Ericaeae), grasses (Poaceae), leguminous plants (Fabaceae), and other herbs, based on Alanen et al. (2011).

Habitat suitability of the release set-aside field was a variable constructed specifically for our current analyses. It was based on extensive quantitative observations on the natural occurrence of the studied butterfly species in the release set-aside field, as explained in Table S1. All the species released in our mobility experiment were classified into three groups: 1 = species never recorded, 2 = species with 1–5 records, and 3 = species with >5 records during years 2003–2011. Class 3 represents species for which the set-aside field was most suitable as a breeding habitat. This measure of habitat suitability was considered as an empirically well-justified and for our purposes more accurate measure of species habitat preference than the previously published classification, presented above.

### Statistical analyses

The first set of statistical analyses focused on mobility differences between two phylogenetically delineated species groups, butterflies (Papilionoidea) and geometroid (Geometroidea) moths (van Nieukerken et al. 2011), using comparably calculated mobility variables as explained above. Noctuoid (Noctuoidea) moths were excluded from these analyses, as there were only a few recaptures (more than one individual recaptured only in one species; Table1).

**Table 1:** Mobility results for all recaptured species: Number of released individuals (*n*), number of recaptured individuals (RC_ind_), recapture probability (%; RC_%_), emigration probability (%; Emig_%_), mean distance moved ± standard error (m; D_mean_ ± SE), and maximum distance moved (m; D_max_). For each butterfly species, the values in parentheses indicate the total number of recaptures and estimates of emigration rate and mean distance moved, based on all recaptures and calculated similarly as in diurnal moths.

Species	*n*	RC_ind_	RC_%_	Emig_%_	D_mean_ ± SE	D_max_
Butterflies						
*Anthocharis cardamines*	22	2 (3)	9.1	100 (100)	985 ± 565 (779 ± 386)	1550
*Aphantopus hyperantus*	188	79 (110)	42.0	14 (10)	113 ± 11 (105 ± 8)	510
*Aricia artaxerxes*	24	5 (6)	20.8	40 (33)	347 ± 119 (310±103)	730
*Boloria euphrosyne*	21	4 (5)	19.0	100 (100)	619 ± 96 (586 ± 81)	893
*Boloria selene*	236	40 (45)	16.9	45 (33)	250 ± 43 (237 ± 38)	885
*Brenthis ino*	92	35 (56)	38.0	51 (41)	147 ± 19 (138 ± 13)	520
*Coenonympha glycerion*	161	25 (34)	15.5	16 (15)	138 ± 21 (132 ± 16)	539
*Gonepteryx rhamni*	44	1	2.3	100	878	878
*Leptidea sinapis*	56	9 (15)	16.1	100 (100)	488 ± 18 (491 ± 12)	548
*Lycaena hippothoe*	33	13 (17)	39.4	8 (12)	84 ± 13 (83 ± 10)	196
*Lycaena virgaureae*	14	6 (8)	42.9	83 (75)	460 ± 71 (430 ± 67)	550
*Melitaea athalia*	21	3	14.3	67	339 ± 241	817
*Nymphalis io*	26	2 (3)	7.7	100 (100)	290 ± 129 (235±92)	419
*Pieris napi*	480	47 (55)	9.8	77 (78)	396 ± 40 (393±35)	1720
*Polyommatus amandus*	253	79 (127)	31.2	19 (20)	119 ± 12 (120±10)	520
*Polyommatus icarus*	25	5 (6)	20.0	40 (33)	191 ± 84 (170 ± 72)	510
*Polyommatus semiargus*	72	15 (17)	20.8	20 (24)	142 ± 34 (137±30)	520
*Thymelicus lineola*	97	15 (17)	15.5	27 (29)	106 ± 13 (112 ± 13)	214
Total	2011^1^	385 (528)	22.2^2^	55.9 (53.9)^2^	338 (315)^2^	
Noctuoid moths						
*Callistege mi*	19	1	5.3	0	34	34
*Cryptocala chardinyi*	16	1	6.3	100	128	128
*Euclidia glyphica*	594	40	6.7	10	122 ± 23	913
Total	673^1^	42	6.1^2^	36.7^2^	95^2^	
Geometroid moths						
*Chiasmia clathrata*	930	41	4.4	0	47 ± 4	130
*Ematurga atomaria*	192	8	4.2	0	64 ± 10	115
*Odezia atrata*	39	1	2.6	100	120	120
*Scotopteryx chenopodiata*	348	11	3.2	0	49 ± 9	107
*Scopula immorata*	38	2	5.3	0	51 ± 1	51
*Siona lineata*	13	2	15.4	0	44 ± 17	61
Total	1694^1^	65	5.9^2^	16.7^2^	63^2^	
Total all	4378^1^	492	16.1^2^	45.1^2^	250^2^	

1 Including also species with no recaptures in the data.

2 Unweighted mean of the recaptured species.

Differences in mean distance moved between the species groups were tested using linear mixed models (LMM) using species group as a categorical fixed factor. Species was included in the model as a random factor in order to take into account the nonindependence of observations from different individuals of the same species. Model fitting was conducted using restricted maximum-likelihood (REML) estimation with the degrees of freedom calculated according to the Kenward–Roger method (Bolker et al. 2009). Differences in emigration rate and recapture probability between the three species groups were tested using the same logic, but by fitting generalized mixed models (GLMM) with logistic link function and binomial error distribution (due to binary response variables). GLMM fitting was conducted using adaptive Gauss–Hermite quadrature estimation (Bolker et al. 2009) with the degrees of freedom calculated with the between–within degrees of freedom approximation. For all three response variables, the pairwise differences between the three species groups were tested using Tukey's test.

As the second step of analyses, multivariate models were built to examine which combinations of species traits best explained mobility differences between butterfly species. Here, only the last recapture of each butterfly individual was taken into account. Also, the sex of each individual was included in these models, because the motivation of the two sexes to move and emigrate may be quite different. However, before multivariate model building, the univariate relationships between each species trait, sex and the three mobility measures were examined by building a separate statistical model for each species trait and mobility measure (Appendix S1). Pairwise relationships between the explanatory species traits were examined before model building in order to avoid inclusion of collinear explanatory variables. Consequently, two species traits (larval host plant type and habitat preference) were omitted from multivariate model building, due to significant relationships with other traits (Appendix S1). Moreover, the potential effect of the original collection area (from the set-aside field or from surrounding landscape) of the released butterfly individuals on mobility was tested, and it did not affect emigration rate or distances moved (Appendix S1). Thus, the role of the source area could be ignored in the analyses.

Forward selection was used in building the LMM and GLMM with multiple variables, that is, the statistically significant variables (*P* < 0.05) were entered into the model in the order of their explanatory power. For the only continuous variable, body size, both linear and quadratic effects were tested. Statistical significances were calculated using an F-test. No overdispersion was observed in the analyses. Pairwise differences between the categories of the categorical species traits were tested using Tukey's test. All LMM and GLMM models described above were built using the statistical package SAS/STAT® 9.2 (SAS institute Inc., Cary, NC).

In order to take into account the potential effects of phylogenetic relatedness on butterfly mobility in our study, the final multivariate models for distance moved and emigration rate were refitted using generalized estimation equations (GEE) as implemented in the ape library, version 3.0.11 (Paradis et al. 2004) in the R statistical environment (R Core Team 2013). GEE are extensions of generalized linear models (GLMs) to be applied when the statistical nonindependence of the data can be determined with a correlation matrix (Paradis and Claude 2002). Paradis and Claude (2002) have demonstrated the applicability of GEE in comparative studies using a between-species correlation matrix derived from a phylogenetic tree, and Pöyry et al. (2009) provide a previous example on butterflies. GEE are especially suitable for data that include categorical variables (Paradis and Claude 2002), as was the case in our study.

To calculate a correlation matrix for relatedness in GEE, a phylogenetic hypothesis was derived for the 32 butterfly species included in our study (Appendix S2). The branching sequences of butterfly families were derived from recent family-level phylogenetic studies covering all higher taxa of butterflies (e.g., Heikkilä et al. 2011). Placement of lower taxa down to individual species was deduced from the phylogenetic studies focusing specifically on each group (Appendix S2). Branches with weak support or unresolved branches in the original studies were treated as polytomies. For simplicity, all tree branches were assumed to be of equal length. In order to include individuals in the analysis, we placed them on species branches so that between-individual distances were assumed to be 0.01 x species branch length. Statistical significances were calculated using an *F*-test, and the phylogenetic hypothesis was used to calculate the corrected degrees of freedom for the data. For recapture probability, the models did not converge using the GEE approach.

## Results

A total of 385 individuals of 18 species of butterflies and 107 individuals of 9 species of moths (6 geometroids and 3 noctuoids) were recaptured within the release set-aside field (328 individuals) and in its surroundings (164 individuals). Table1 summarizes information on the released and recaptured individuals and their mobility for all species with at least one recapture (for information on all released species, see Table S1).

### Differences between butterflies and moths

**Figure 2:**
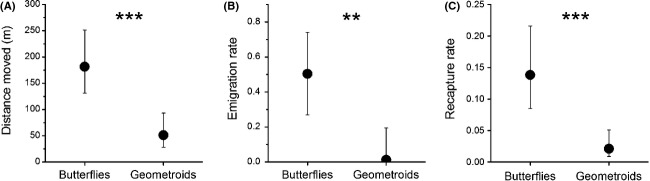
Differences in (A) mean distance moved, (B) emigration rate, and (C) recapture rate between butterflies and geometroid moths. Means are least squares means (LSM) with 95% confidence intervals based on the statistical models fitted to collected data (Table2). The asterisks indicate the statistical difference between the species groups (**P < 0.01, ***P < 0.001).

The two compared species groups, butterflies and geometroid moths, differed significantly in all three examined measures of mobility (Table2, Fig.2). Butterflies were more mobile than geometroids as indicated by their longer mean distances moved (315 m vs. 63 m) and higher emigration rate (54% vs. 17%). The higher recapture rate of butterflies than geometroids (22% vs. 6%) most probably reflected the better detectability in butterflies than geometroids. The mobility of noctuoid moths seemed to be somewhere between butterflies and geometroids (Table1), but the noctuoid data were too limited to allow meaningful statistical analyses.

**Table 2:** LMM and GLMM results on the differences in the three mobility variables between butterflies and geometroid moths. The differences between the species groups remained significant in all three variables when the models were refitted for the subset of species for which the release set-aside provided suitable habitat (release habitat suitability class = 3).

Response variable	Model	*n*	df (numerator:denominator)	*F*	*P*
Distance moved	LMM	593	1:23.2	14.71	0.0008
Emigration rate	GLMM	593	2:22	8.22	0.0090
Recapture rate	GLMM	3848	2:52	16.69	0.0002

### Butterfly movements in relation to species traits

The studied butterfly species showed a lot of interspecific variation in mobility. Average distance moved varied from 84 m and 106 m in the two most sedentary species (*Lycaena hippothoe* and *Thymelicus lineola*, respectively) to 619 m and 985 m in the two most mobile species (*Boloria euphrosyne* and *Anthocharis cardamines*, respectively). Emigration from the release set-aside field varied from 8% (*L. hippothoe*) and 14% (*Aphantopus hyperantus*) to 100% in five of the studied species (Table1).

**Figure 3:**
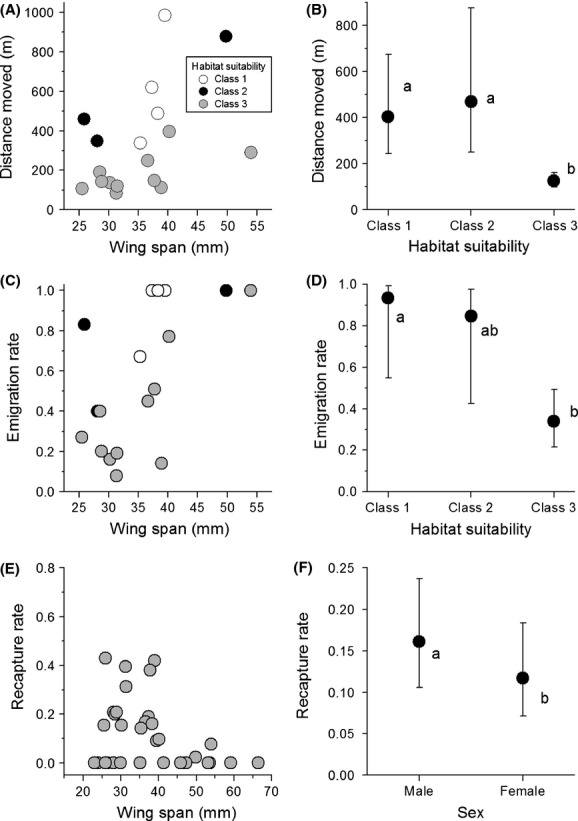
Statistically significant relationships between species traits and the three mobility variables: (A–B) distance moved, (C–D) emigration rate, and (E–F) recapture rate in butterflies. Means are least squares means (LSM) with 95% confidence intervals based on the multivariate models fitted to collected data (Table3). In the panels A, C, and E, the dots represent means for individual species. The letters a and b within the panels B, D, and F indicate homogeneous groups and thus the treatments which differed significantly in pairwise comparisons.

Two species traits, release habitat suitability and body size, became included together in the multivariate models best explaining the two main mobility variables, distance moved (LMM) and emigration rate (GLMM) (Table3A). The effects of the two traits were very similar in both models. Distance moved and emigration rate were lower in butterfly species for which habitat suitability was the highest. Furthermore, both distance moved and emigration rate tended to increase with increasing body size, when the effect of habitat suitability was taken into account (see also Fig.3A and C).

**Table 3:** Results of the final multivariate models (LMM, GLMM, and GEE) for the three studied mobility variables. (A) LMM and GLMM models without accounting for the phylogenetic relatedness. (B) GEE models accounting for the phylogenetic relatedness among species

Model^2^	Estimate ± SE	df	*F*	*P*
(A)				
Distance moved (LMM)				
Constant	3.354 ± 0.589			
Hab_suit_		2:30.5	15.57	<0.001
Class 1	1.168 ± 0.278			
Class 2	1.316 ± 0.325			
Body size	0.042 ± 0.017	1:27.8	6.21	0.019
Emigration rate (GLMM)				
Constant	−5.517 ± 1.850			
Hab_suit_		2:14	6.76	0.009
Class 1	3.334 ± 1.195			
Class 2	2.384 ± 0.967			
Body size	0.139 ± 0.054	1:14	6.70	0.022
Recapture rate (GLMM)				
Constant	12.468 ± 4.301			
Body size	0.605 ± 0.238	1:29	6.48	0.017
Body size^*^Body size	−0.009 ± 0.003	1:29	7.08	0.013
Sex		1:22	6.32	0.020
*Male*	0.372 ± 0.148			
(B)				
Distance moved (GEE)^3^				
Hab_suit_[Table-fn tf3-4]	−0.729 ± 0.186	2	29.43	0.011
Body size	0.039 ± 0.020	1	3.95	0.141
Emigration rate (GEE)^3^				
Hab_suit_^4^	−1.315 ± 0.739	2	13.99	0.030
Body size	0.141 ± 0.028	1	25.19	0.015

Hab_suit_ = release habitat suitability (Class 1 = unsuitable, Class 2 = fairly unsuitable, Class 3 = suitable habitat for breeding), Body size = wingspan (mm).

2 GEE Model for recapture probability did not converge.

3 Phylogenetic degrees of freedom: 7.00.

4 Habitat suitability (Hab_suit_) was treated as an ordered factor in both models, and model estimates for linear contrasts are presented in the table.

For the third mobility variable, recapture rate, body size, and sex were the two variables included together in the multivariate GLMM (Table3A). The effect of body size became significant only when its nonlinear component was included in the model. Recapture rate was highest in butterfly species of intermediate size and particularly low in the largest species released in the experiment. The significant effect of sex was due to the higher recapture rate of males than females.

When the final LMM and GLMM models were refitted using generalized estimation equations (GEE) in order to take into account the potential effects of phylogenetic relatedness, the results changed slightly (Table3B). In the GEE model for distance moved, the effect of body size did not remain significant (*P* = 0.14), but habitat suitability still had a significant effect. In the GEE model for emigration rate, both habitat suitability and body size had a significant effect.

## Discussion

The release experiment successfully produced directly comparable mobility data for butterflies and moths. Recaptures were collected from almost 500 individuals belonging to 27 species. The data set enabled us both to detect differences in mobility between two lepidopteran superfamilies and to identify significant effects of species traits on distance moved and emigration rate in 18 species of butterflies.

### Differences between butterflies and moths

As expected, experimentally released butterflies were more mobile than thin-bodied, weakly flying geometroid moths in terms of both distance moved and emigration rate. Butterfly movement distances were on the average five times longer and emigration rate three times higher than in geometroid moths. Data for noctuoid moths remained too sparse to infer any general results. Our findings are in agreement with our previous results on colonization of set-asides by butterflies and diurnal moths (Alanen et al. 2011) and an experiment comparing mobility of lepidopteran species groups (Nieminen 1996) in a network of small islands. Like our results, the results of Nieminen also suggested that butterflies are most and thin-bodied geometroids least mobile, whereas noctuoids show intermediate mobility. It should be noted, however, that Nieminen studied only two butterfly species, *Vanessa atalanta* and *Hipparchia semele*, of which *V. atalanta* is known as a regular long-distance migrant, representing one of the most mobile butterfly species occurring in Europe (Stefanescu 2001).

Recapture rate was generally much lower in diurnal moths than in butterflies. We argue that there are two likely reasons for this: the lower flight activity and thus the lower detectability of moths and the higher population densities of the most abundant moths compared to the most abundant butterflies. Based on the observed relative abundances of marked vs. unmarked individuals in the release set-aside field, we estimated that the geometroid *Semiothisa clathrata* and the noctuoid *Euclidia glyphica*, for instance, were an order of magnitude more abundant than the most abundant butterflies, such as *Aphantopus hyperantus* and *Polyommatus amandus*. Nevertheless, due to our systematic sampling protocol, the relative recapture probabilities of the studied taxonomic groups did not differ at different distances from the release point, and thus the mobility results can be reliably compared between different species and species groups. Our results indicate that it is more difficult to obtain reliable mobility data from diurnal moths than butterflies by mark–release–recapture method.

In light of the theoretical model by Travis and Dytham (1999), the observed pattern of mobility variation across moth and butterfly species in our experiment has potential consequences for species persistence. According to their predictions, species with either low or high dispersal rate should perform best in highly fragmented landscapes, whereas species with intermediate mobility are predicted to perform worst. Our findings seem to fit these predictions because the geometroid moths, that were found to be the least mobile lepidopterans, have not declined in Finland (Huldén et al. 2000) and are typically common and abundant in many kinds of uncultivated grassland. Similarly, large butterfly species with high mobility have not suffered from habitat fragmentation, whereas some grassland specialist butterflies with intermediate mobility, such as *L. hippothoe*, have disappeared from many intensively cultivated landscapes (Ekroos and Kuussaari 2012). This model prediction has previously received empirical support from British butterflies (Thomas 2000).

### Butterfly movements in relation to species traits

Butterfly mobility was strongly affected by habitat suitability. Butterflies tended to quickly emigrate from the release set-aside field, if it did not offer suitable breeding habitat for the species in question. Body size explained additional variation in mobility after the effect of habitat suitability had been taken into account in the statistical models. Both distance moved and emigration rate increased with body size, as expected based on our earlier results on butterfly colonization speed (Alanen et al. 2011) and meta-analyses on butterfly mobility (Stevens et al. 2010, 2012; Sekar 2012). When phylogenetic relatedness among species was included in the analyses, the significant effect of body size disappeared for distance moved, but habitat suitability remained significant.

#### Habitat suitability

Comparison of average emigration rates in terms of habitat suitability highlights its importance in butterfly mobility: On the average, 33% of recaptured individuals had emigrated in those species that naturally occurred in the release set-aside, whereas 94% of individuals had emigrated in species for which the set-aside was considered unsuitable for breeding. The observed emigration rates in grassland species, for which the release set-aside field provided suitable breeding habitat, are roughly similar to previous observations on grassland specialist butterfly metapopulations (Hovestadt and Nieminen 2009; Stevens et al. 2010). The systematically high emigration rate in species, for which the set-aside was unsuitable for breeding, can be understood as a natural dispersal response owing to their unfitting habitat preference (mostly for forest edges and clearings, Table S2), lack of required larval host plants, and consequently, lack of conspecific individuals within the release set-aside field. Previously Conradt et al. (2001) have shown that individuals of *Pyronia tithonus* exhibited distinctly different flight behavior when released in an unsuitable compared with a suitable breeding habitat.

Even though the important role of release habitat suitability was not surprising, we could not find any previous studies which would have empirically documented it across multiple species. This is probably due to the difficulty of directly detecting habitat suitability effects on mobility without experimentally manipulating butterfly occurrence. Previous experimental studies examining butterfly flight behavior by releasing individuals in field conditions have typically focused only on some components of flight or dispersal behavior (Conradt et al. 2001; Ries and Debinski 2001; Söderström and Hedblom 2007; Schultz et al. 2012) and have not specifically studied mobility differences across several species at a large spatial scale. In this regard, the recent study by Kallioniemi et al. (2014) is exceptional, because they examined butterfly behavior at habitat boundaries in a release experiment and reported differences in the likelihood of crossing habitat boundaries in seven butterfly species.

Our results indicate that butterflies recognize suitable habitats during dispersal and may switch to more sedentary behavior when encountering them. Species preferring forest edges showed a high emigration rate, and several individuals were recaptured in the only relatively nearby forest edge habitat, at *c*. 600 m distance from the release set-aside field (Fig.1). However, it is unlikely that butterflies could have visually recognized the forest edge already from the release set-aside, as previous studies suggest that distances from which butterflies are capable of recognizing suitable habitat are much shorter. For example, Conradt et al. (2001) released individuals of two butterfly species within unsuitable habitat at different distances from a suitable habitat patch and found that *Maniola jurtina* and *Pyronia tithonus* were usually capable of locating the suitable habitat at 65–85 m distance but not further away from the release point.

#### Body size

The finding of a positive relationship between butterfly body size and mobility was expected and in agreement with the meta-analyses by Stevens et al. (2010) and Sekar (2012), even though our results probably underestimated the significance of body size owing to the very low number of recaptures in the largest species. These species, such as *Nymphalis urticae* (no recaptures), *N. io* (2 recaptures), and large fritillaries in the genus *Argynnis* (no recaptures), are strong and fast fliers and thus difficult to catch in the field (see Fig.3E and Table S1). More recaptures from these species would probably have strengthened the correlation between mobility and body size. Residual variance of the body size–mobility relationship was largely explained by release habitat suitability. This finding is in agreement with the results of Stevens et al. (2012) who concluded that even though butterfly body size seems to always be positively correlated with measures of mobility, its predictive power is limited without taking other key species traits into account.

In addition, we found phylogeny to play an important role in butterfly mobility, which is in contrast with Stevens et al. (2012). The effect of body size on distance moved did not remain significant after the phylogenetic relatedness of butterfly species had been taken into account. This is not surprising, as a substantial proportion of variation in body size between butterfly species stems directly from size differences between butterfly families (e.g., Nymphalidae vs. Lycaenidae), whereas size differences are often small between closely related species within a family (e.g., within Lycaenidae and Hesperiidae).

## Conclusions

Our release experiment showed that comparable multispecies data on important components of insect mobility can be gathered simultaneously at a relatively large spatial scale. Three conclusions can be drawn based on the results. First, butterflies moved longer distances and had higher emigration rate than geometroid moths. Second, release habitat suitability had a strong effect on butterfly mobility so that species naturally occurring in the release set-aside were much less mobile than species for which the set-aside was not a suitable breeding habitat. Third, mobility of butterflies increased significantly with body size after the effect of habitat suitability had been taken into account, but the effect of body size was partly confounded by phylogenetic relatedness. The experimental multispecies approach used here offers interesting opportunities for future studies of insect mobility. It builds on the tradition of studying mobility and dispersal behavior based on experimental releases of individuals, but which previously have focused at only one or a few species and conducted at smaller spatial scale (see Kallioniemi et al. 2014 and references therein for recent examples).

## Data accessibility

All relevant information on the analyzed mobility measures and species traits are given for all the studied species in the electronic Supporting information of this paper.

## Supporting Information

**Appendix S1.** Univariate models and correlations between species traits. **Appendix S2.** Phylogenetic hypothesis for the butterfly species in the study. **Table S1.** Experimentally released species with information on their natural occurrence in the release set-aside field. **Table S2.** Butterfly species traits used in the mobility analyses.
