# Supplementary material for: Higher mobility of butterflies than moths connected to habitat suitability and body size in a release experiment
Source: Ecol Evol. 2014 Sep 12;4(19):3800–11. doi: 10.1002/ece3.1187 (PMC4301046; doi:10.1002/ece3.1187)
Supplement: Supplementary file 1 — Appendix S1. Univariate models and correlations between species traits. Appendix S2. Phylogenetic hypothesis for the butterfly species in the study. Table S1. Experimentally released species with information on their natural occurrence in the release set-aside field. Table S2. Butterfly species traits used in the mobility analyses. [file ece30004-3800-sd1.docx]

**Supporting Information**

for the paper “Higher mobility of butterflies than moths connected to habitat suitability and body size in a release experiment” by Mikko Kuussaari, Matias Saarinen, Eeva-Liisa Korpela, Juha Pöyry & Terho Hyvönen

**Appendix S1.** Univariate models and correlations between species traits (page 2)

**Appendix S2.** Phylogenetic hypothesis for the butterfly species in the study (page 5)

**Table S1.** Experimentally released species with information on their natural occurrence in the release set-aside field (page 10)

**Table S2.** Butterfly species traits used in the mobility analyses (page 12)

**Appendix S1.** *Univariate models and correlations between species traits*

**Methods**

The univariate relationships between each species trait, sex and the three mobility measures were examined by building a separate statistical model for each species trait and mobility measure. LMMs were used to analyse the role of species traits in mean distance moved and GLMMs to analyse their role in emigration rate and recapture probability. Species was always included as a random factor. Pairwise differences between the categories of the categorical species traits were tested using Tukey’s test.

In a similar way the potential effect of the source (collection) area of the released butterfly individuals on the three mobility variables was tested for the subset of released species (*n* = 9),in which at least one individual originated both from the release fallow field and its surroundings.

Pairwise relationships between body size and the categorical species traits were tested using analysis of variance (ANOVA) and the relationships between the categorical variables using contingency tables.

**Results**

*Univariate models*

In the univariate LMMs, two species traits, release habitat suitability and habitat preference, had a significant effect on mean distance moved (Table 1). Distances moved were especially high in species for which the release set-aside field was not a suitable habitat and in species which prefer forest edges as their main breeding habitat. Distances moved were significantly shorter in species that naturally occurred in the set-aside field and in species preferring grasslands. Distance moved also increased marginally significantly with body size (Table 1).

Emigration rate was rather similarly affected by habitat suitability and habitat preference as were distances moved. Emigration probability was high in species for which the release set-aside field was not a suitable habitat as well as in forest edge or field margin species and significantly lower in species that naturally occurred in the set-aside field as well as in grassland species. Unlike in distance moved, however, emigration rate increased significantly with butterfly body size in the univariate GLMM (Table 1).

For recapture probability, statistically significant effects of species traits were somewhat different than in distance moved and emigration rate (Table 1). Habitat preference had a strong effect, recapture rate being much higher in grassland than in forest edge species. Body size had a unimodal effect on recapture probability with the highest recapture rates in species of intermediate size. Sex also had a significant effect, males having on the average a higher recapture rate than females. However, this difference between the sexes was not systematic across different species (*χ²* = 11.5, *P* > 0.5), and in some species the recapture rate was higher in females. In addition, larval host plant type had a marginally significant effect, as species feeding on forest plants tended to have the lowest recapture rate.

*Role of butterfly source area*

Source area (release fallow or its surroundings) of the released individuals did not affect mean distance moved or emigration probability in the subset of data in which the testing was possible (*P*-values 0.50 and 0.59, respectively; Table 2). However, original source area affected recapture probability, which was significantly higher (*P* = 0.03) in individuals originally captured from the release fallow field (0.22) than from its surroundings (0.15; Table 2).

*Correlations between species traits*

Two species traits, larval host plant type and habitat preference, were omitted from multivariate model building, due to significant correlations (Table 3). Habitat preference was omitted because it was significantly associated with both body size and release habitat suitability. Larval host plant type was omitted because it was significantly associated with release habitat suitability as well as with habitat preference.

**Table 1.** Results of the univariate models for variables explaining butterfly mobility in LMMs and GLMMs. Hab_suit_ = Habitat suitability, Hab_pref_ = Habitat preference, Hab_spec_ = Habitat specificity, Host_pref_ = Larval host plant type, Host_spec_ = Larval host plant specificity.

| **Response variable**  Explanatory variable | **d.f. (numerator:denominator)** | ***F*** | ***P*** | **Deviance** | **AIC_c_** |
| --- | --- | --- | --- | --- | --- |
| **Distance moved** (*n* = 385, LMM) | |  |  |  |  |
| Size | 1:20.6 | 3.45 | 0.0777 | 906.0 | 910.0 |
| Hab_suit_ | 2:24.4 | 13.09 | 0.0001 | 886.7 | 890.8 |
| Hab_pref_ | 2:12.3 | 12.47 | 0.0011 | 888.0 | 892.0 |
| Hab_spec_ | 1:15.0 | 1.55 | 0.2322 | 902.4 | 906.5 |
| Host_pref_ | 3:16.8 | 2.38 | 0.1062 | 895.1 | 899.1 |
| Host_spec_ | 1:14.5 | 0.06 | 0.8058 | 903.9 | 907.9 |
| Sex | 1:371 | 1.63 | 0.2031 | 905.0 | 909.0 |
| **Emigration rate** (*n* = 385, GLMM) | |  |  |  |  |
| Size | 1:16 | 5.23 | 0.0361 | 423.8 | 429.8 |
| Hab_suit_ | 2:15 | 5.67 | 0.0147 | 416.6 | 424.7 |
| Hab_pref_ | 2:15 | 9.91 | 0.0018 | 410.2 | 418.3 |
| Hab_spec_ | 1:16 | 1.15 | 0.2988 | 428.8 | 434.8 |
| Host_pref_ | 3:14 | 2.17 | 0.1367 | 423.2 | 4334 |
| Host_spec_ | 1:16 | 0.14 | 0.7172 | 429.7 | 435.8 |
| Sex | 1:13 | 1.06 | 0.3227 | 428.8 | 434.9 |
| **Recapture rate** (*n* = 2011, GLMM) | |  |  |  |  |
| Size +  Size*Size | 1:29 | 6.28  6.90 | 0.0181  0.0136 | 1819.3 | 1827.4 |
| Hab_suit_ | 1:29 | 2.31 | 0.1172 | 1823.2 | 1831.3 |
| Hab_pref_ | 2:28 | 8.47 | 0.0013 | 1813.3 | 1821.3 |
| Hab_spec_ | 1:30 | 0.02 | 0.8777 | 1827.8 | 1833.8 |
| Host_pref_ | 3:27 | 2.87 | 0.0551 | 1815.5 | 1825.5 |
| Host_spec_ | 1:30 | 1.02 | 0.3211 | 1826.8 | 1832.8 |
| Sex | 1:22 | 6.12 | 0.0216 | 1821.5 | 1827.5 |

**Table 2.** Results of univariate LMM and GLMMs testing the effect of the source (collection) area (release fallow field or its surroundings) of the released butterflies on the three mobility variables.

| Response variable | Model | *n* | d.f.  (numerator:denominator) | *F* | *P* |
| --- | --- | --- | --- | --- | --- |
| Distance moved | LMM | 313 | 1:8 | 0.51 | 0.496 |
| Emigration rate | GLMM | 313 | 1:8 | 0.31 | 0.594 |
| Recapture rate | GLMM | 480 | 1:8 | 6.85 | 0.031 |

**Table 3.** Statistical relationships between the studied species traits in butterflies.

|  | **Size^(1^** | **Hab_suit_^(2^** | **Hab_pref_^(2^** | **Hab_spec_^2^** | **Host_pref_^(2^** |
| --- | --- | --- | --- | --- | --- |
| **Hab_suit_** | *F*_2,60_=1.965  *P*=0,15 | |  |  |  |
| **Hab_pref_** | *F*_2,54_=5,215 *P*=0.009 | *P*=0.000 |  |  |  |
| **Hab_spec_** | *F*_1,31_=1,219 *P*=0.279 | *P*=0.270 | *P*=0.043 |  |  |
| **Host_pref_** | *F*_3,54_=0,736 *P*=0.535 | *P*=0.006 | *P*=0.000 | *P*=0.574 |  |
| **Host_spec_** | *F*_1,54_=1,192 *P*=0.280 | *P*=0.110 | *P*=0.915 | *P*=0.242 | *P*=0.118 |

ANOVA^(1^ and Fisher’s exact^(2^

**Appendix S2**. *Phylogenetic hypothesis for the butterfly species in the study*

**Description of the methods and literature used in building the phylogenetic hypothesis for the butterfly species included in the study**

After constructing the overall relationships among the butterfly families following Heikkilä *et al.* ([2011](#_ENREF_5)) and Regier *et al.* (2013), we continued by placing lower taxa (subfamilies, tribes, genera and species) on the tree. This was done on the basis of the phylogenetic studies focusing specifically on each butterfly group.

For the Hesperiidae part of the phylogeny, we consulted Warren *et al.* ([2008](#_ENREF_24); [2009](#_ENREF_25)); for Pieridae we consulted Braby *et al.* ([2006](#_ENREF_2)), Chew & Watt ([2006](#_ENREF_3)) and Wheat & Watt ([2008](#_ENREF_26)); for Lycaenidae we consulted Als *et al.* ([2004](#_ENREF_1)), Fric *et al.* ([2007](#_ENREF_4)), Wiemers *et al.* ([2010](#_ENREF_27)), Ugelvig *et al.* ([2011](#_ENREF_17)), Vila *et al.* ([2011](#_ENREF_28)), and Talavera *et al.* ([2013](#_ENREF_16)) (Note that all the Lycaenidae studies focus on the subfamily Polyommatinae, whereas there are no studies on subfamilies Lycaeninae and Theclinae); For positions of different subfamilies within the family Nymphalidae we consulted Wahlberg *et al.* ([2003](#_ENREF_23); [2009](#_ENREF_20)); for the subfamily Heliconiinae we consulted Simonsen *et al.* ([2006](#_ENREF_13); [2010](#_ENREF_14)); for the subfamily Satyrinae we consulted Martin *et al.* ([2000](#_ENREF_9)), Peña *et al.* ([2006](#_ENREF_11)), Peña & Wahlberg ([2008](#_ENREF_10)), Kodandaramaiah & Wahlberg ([2009](#_ENREF_7)) and Kodandaramaiah *et al.* ([2010](#_ENREF_6)); and for the subfamily Nymphalinae we consulted Wahlberg & Nylin ([2003](#_ENREF_21)), Wahlberg *et al.* ([2005](#_ENREF_19)), Wahlberg ([2006](#_ENREF_18)) and Wahlberg & Rubinoff ([2011](#_ENREF_22)).

Branches with weak statistical support (i.e. unresolved branches) in the original studies were treated as polytomies in the phylogenetic hypothesis derived by us. For taxa with no phylogenetic studies available yet, we applied the phylogenetic subtraction method described by Stearns ([1983](#_ENREF_15)). In this method species are placed on a phylogenetic tree based on the contemporarily accepted taxonomical categories. For this purpose we used primarily the list of Finnish Lepidoptera that covers all butterfly species observed in the study (Kullberg *et al.* 2008).

The phylogenetic hypothesis used in the analyses of distances moved and emigration rate is shown in Fig. 2. and that used in the analysis of recapture rate in Fig. 2.

**Fig. 1**. Phylogenetic hypothesis for the 18 butterfly species included in the GEE analyses of distance moved and emigration rate.

**Fig. 2**. Phylogenetic hypothesis for the 32 butterfly species included in the GEE analysis of recapture rate.

**References**

Als, T.D., Vila, R., Kandul, N.P., Nash, D.R., Yen, S.-H., Hsu, Y.-F., Mignault, A.A., Boomsma, J.J. & Pierce, N.E. (2004) The evolution of alternative parasitic life histories in large blue butterflies. *Nature*, **432**, 386-390.

Braby, M.F., Vila, R. & Pierce, N.E. (2006) Molecular phylogeny and systematics of the Pieridae (Lepidoptera: Papilionoidea): higher classification and biogeography. *Zoological Journal of the Linnean Society*, **147**, 239-275.

Chew, F.S. & Watt, W.B. (2006) The green-veined white (*Pieris napi* L.), its Pierinae relatives, and the systematics dilemmas of divergent character sets (Lepidoptera, Pieridae). *Biological Journal of the Linnean Society*, **88**, 413-435.

Fric, Z., Wahlberg, N., Pech, P. & Zrzavý, J.A.N. (2007) Phylogeny and classification of the *Phengaris–Maculinea* clade (Lepidoptera: Lycaenidae): total evidence and phylogenetic species concepts. *Systematic Entomology*, **32**, 558-567.

Heikkilä, M., Kaila, L., Mutanen, M., Peña, C. & Wahlberg, N. (2011) Cretaceous origin and repeated tertiary diversification of the redefined butterflies. *Proceedings of the Royal Society B: Biological Sciences*, **279**, 1093-1099.

Kodandaramaiah, U., Peña, C., Braby, M.F., Grund, R., Müller, C.J., Nylin, S. & Wahlberg, N. (2010) Phylogenetics of Coenonymphina (Nymphalidae: Satyrinae) and the problem of rooting rapid radiations. *Molecular Phylogenetics and Evolution*, **54**, 386-394.

Kodandaramaiah, U. & Wahlberg, N. (2009) Phylogeny and biogeography of *Coenonympha* butterflies (Nymphalidae: Satyrinae) - patterns of colonization in the Holarctic. *Systematic Entomology*, **34**, 315-323.

Kullberg, J., Albrecht, A., Kaila, L. & Varis, V. (2008) Checklist of Finnish Lepidoptera - an updated version. Finnish Museum of Natural History, Helsinki [http://www.luomus.fi/elaintiede/hyonteiset/perhoset/, Accessed on 24 September, 2013].

Martin, J.F., Gilles, A. & Descimon, H. (2000) Molecular Phylogeny and Evolutionary Patterns of the European Satyrids (Lepidoptera: Satyridae) as Revealed by Mitochondrial Gene Sequences. *Molecular Phylogenetics and Evolution*, **15**, 70-82.

Peña, C. & Wahlberg, N. (2008) Prehistorical climate change increased diversification of a group of butterflies. *Biology Letters*, **4**, 274-278.

Peña, C., Wahlberg, N., Weingartner, E., Kodandaramaiah, U., Nylin, S., Freitas, A.V.L. & Brower, A.V.Z. (2006) Higher level phylogeny of Satyrinae butterflies (Lepidoptera: Nymphalidae) based on DNA sequence data. *Molecular Phylogenetics and Evolution*, **40**, 29-49.

Regier, J.C., Mitter, C., Zwick, A., Bazinet, A.L., Cummings, M.P., Kawahara, A.Y., Sohn, J.-C., Zwickl, D.J., Cho, S., Davis, D.R., Baixeras, J., Brown, J., Parr, C., Weller, S., Lees, D.C. & Mitter, K.T. (2013) A Large-Scale, Higher-Level, Molecular Phylogenetic Study of the Insect Order Lepidoptera (Moths and Butterflies). *PLoS ONE*, **8**, e58568.

Simonsen, T.J., Wahlberg, N., Brower, A.V.Z. & de Jong, R. (2006) Morphology, molecules and fritillaries: approaching a stable phylogeny for Argynnini (Lepidoptera: Nymphalidae). *Insect Systematics & Evolution*, pp. 405-418.

Simonsen, T.J., Wahlberg, N., Warren, A.D. & Sperling, F.A.H. (2010) The evolutionary history of *Boloria* (Lepidoptera: Nymphalidae): phylogeny, zoogeography and larval–foodplant relationships. *Systematics and Biodiversity*, **8**, 513-529.

Stearns, S.C. (1983) The influence of size and phylogeny on patterns of covariation among life history traits in mammals. *Oikos*, **41**, 173-187.

Talavera, G., Lukhtanov, V.A., Pierce, N.E. & Vila, R. (2013) Establishing criteria for higher-level classification using molecular data: the systematics of *Polyommatus* blue butterflies (Lepidoptera, Lycaenidae). *Cladistics*, **29**, 166-192.

Ugelvig, L.V., Vila, R., Pierce, N.E. & Nash, D.R. (2011) A phylogenetic revision of the *Glaucopsyche* section (Lepidoptera: Lycaenidae), with special focus on the *Phengaris–Maculinea* clade. *Molecular Phylogenetics and Evolution*, **61**, 237-243.

Wahlberg, N. (2006) That awkward age for butterflies: Insights from the age of the butterfly subfamily Nymphalinae (Lepidoptera: Nymphalidae). *Systematic Biology*, **55**, 703-714.

Wahlberg, N., Brower, A.V.Z. & Nylin, S. (2005) Phylogenetic relationships and historical biogeography of tribes and genera in the subfamily Nymphalinae (Lepidoptera: Nymphalidae). *Biological Journal of the Linnean Society*, **86**, 227-251.

Wahlberg, N., Leneveu, J., Kodandaramaiah, U., Peña, C., Nylin, S., Freitas, A.V.L. & Brower, A.V.Z. (2009) Nymphalid butterflies diversify following near demise at the Cretaceous/Tertiary boundary. *Proceedings of the Royal Society B: Biological Sciences*, **276**, 4295-4302.

Wahlberg, N. & Nylin, S. (2003) Morphology versus molecules: resolution of the positions of *Nymphalis*, *Polygonia*, and related genera (Lepidoptera: Nymphalidae). *Cladistics*, **19**, 213-223.

Wahlberg, N. & Rubinoff, D. (2011) Vagility across *Vanessa* (Lepidoptera: Nymphalidae): mobility in butterfly species does not inhibit the formation and persistence of isolated sister taxa. *Systematic Entomology*, **36**, 362-370.

Wahlberg, N., Weingartner, E. & Nylin, S. (2003) Towards a better understanding of the higher systematics of Nymphalidae (Lepidoptera: Papilionoidea). *Molecular Phylogenetics and Evolution*, **28**, 473-484.

Warren, A.D., Ogawa, J.R. & Brower, A.V.Z. (2008) Phylogenetic relationships of subfamilies and circumscription of tribes in the family Hesperiidae (Lepidoptera: Hesperioidea). *Cladistics*, **24**, 642-676.

Warren, A.D., Ogawa, J.R. & Brower, A.V.Z. (2009) Revised classification of the family Hesperiidae (Lepidoptera: Hesperioidea) based on combined molecular and morphological data. *Systematic Entomology*, **34**, 467-523.

Wheat, C.W. & Watt, W.B. (2008) A mitochondrial-DNA-based phylogeny for some evolutionary-genetic model species of *Colias* butterflies (Lepidoptera, Pieridae). *Molecular Phylogenetics and Evolution*, **47**, 893-902.

Wiemers, M., Stradomsky, B.V. & Vodolazhsky, D.I. (2010) A molecular phylogeny of *Polyommatus* s. str. and *Plebicula* based on mitochondrial COI and nuclear ITS2 sequences (Lepidoptera: Lycaenidae). *European Journal of Entomology*, **107**, 325-336.

Vila, R., Bell, C.D., Macniven, R., Goldman-Huertas, B., Ree, R.H., Marshall, C.R., Balint, Z., Johnson, K., Benyamini, D. & Pierce, N.E. (2011) Phylogeny and palaeoecology of *Polyommatus* blue butterflies show Beringia was a climate-regulated gateway to the New World. *Proceedings of the Royal Society B: Biological Sciences*, **278**, 2737-2744.

**Table S1.** Experimentally released species with information on their natural occurrence in the release set-aside field. The first two columns *n* and RC_ind_ indicate the total number of released and recaptured individuals, respectively. The next seven columns indicate the total number of individuals recorded in the release set-aside field in standardized transect counts during 2003-2008 (Alanen *et al.* 2011) and in additional, less systematic counts made during the release experiment in summer 2011. Hab_suit_ indicates the habitat suitability class for each species: 1 = species never recorded (i.e. species for which the set-aside field was the least suitable habitat), 2 = species with 1-5 records and 3 = species with >5 records (species for which the set-aside field was the most suitable habitat).

| **Species** | **Release experiment** | | **Natural occurrence in the release set-aside field** | | | | | | | | **Hab_suit_** |
| --- | --- | --- | --- | --- | --- | --- | --- | --- | --- | --- | --- |
|  | ***n*** | **RC_ind_** | **2003** | **2004** | **2005** | **2006** | **2007** | **2008** | **2011*** | **Total** |  |
| **Butterflies** |  |  |  |  |  |  |  |  |  |  |  |
| *Anthocharis cardamines* | 22 | 2 | 0 | 0 | 0 | 0 | 0 | 0 | 0 | 0 | 1 |
| *Aphantopus hyperantus* | 188 | 79 | 8 | 34 | 44 | 268 | 336 | 357 | 36 | 1083 | 3 |
| *Araschnia levana* | 1 | 0 | 0 | 0 | 0 | 0 | 0 | 0 | 0 | 0 | 1 |
| *Argynnis adippe* | 4 | 0 | 0 | 0 | 1 | 1 | 0 | 0 | 0 | 2 | 2 |
| *Argynnis aglaja* | 1 | 0 | 0 | 0 | 0 | 0 | 0 | 0 | 1 | 1 | 2 |
| *Aricia artaxerxes* | 24 | 5 | 0 | 0 | 0 | 0 | 0 | 0 | 3 | 3 | 2 |
| *Boloria euphrosyne* | 21 | 4 | 0 | 0 | 0 | 0 | 0 | 0 | 0 | 0 | 1 |
| *Boloria selene* | 236 | 40 | 0 | 1 | 2 | 46 | 6 | 0 | 1 | 56 | 3 |
| *Brenthis ino* | 92 | 35 | 0 | 1 | 12 | 13 | 7 | 0 | 29 | 62 | 3 |
| *Callophrys rubi* | 39 | 0 | 0 | 0 | 0 | 0 | 0 | 0 | 0 | 0 | 1 |
| *Celastrina argiolus* | 15 | 0 | 0 | 0 | 0 | 0 | 0 | 0 | 0 | 0 | 1 |
| *Coenonympha glycerion* | 161 | 25 | 0 | 3 | 3 | 54 | 79 | 16 | 77 | 232 | 3 |
| *Erebia ligea* | 1 | 0 | 0 | 0 | 0 | 0 | 0 | 1 | 0 | 1 | 2 |
| *Aricia eumedon* | 31 | 0 | 0 | 0 | 1 | 0 | 0 | 0 | 0 | 1 | 2 |
| *Gonepteryx rhamni* | 44 | 1 | 0 | 0 | 1 | 1 | 0 | 0 | 1 | 3 | 2 |
| *Leptidea sinapis* | 56 | 9 | 0 | 0 | 0 | 0 | 0 | 0 | 0 | 0 | 1 |
| *Lycaena hippothoe* | 33 | 13 | 0 | 0 | 0 | 0 | 0 | 0 | 21 | 21 | 3 |
| *Lycaena virgaureae* | 14 | 6 | 0 | 0 | 2 | 0 | 0 | 0 | 0 | 2 | 2 |
| *Melitaea athalia* | 21 | 3 | 0 | 0 | 0 | 0 | 0 | 0 | 0 | 0 | 1 |
| *Nymphalis antiopa* | 1 | 0 | 0 | 0 | 0 | 0 | 0 | 0 | 0 | 0 | 1 |
| *Nymphalis io* | 26 | 2 | 0 | 1 | 174 | 24 | 0 | 0 | 21 | 220 | 3 |
| *Nymphalis urticae* | 13 | 0 | 9 | 11 | 36 | 65 | 20 | 4 | 27 | 172 | 3 |
| *Ochlodes sylvanus* | 28 | 0 | 0 | 5 | 14 | 29 | 16 | 0 | 2 | 66 | 3 |
| *Pieris napi* | 480 | 47 | 9 | 0 | 119 | 29 | 6 | 8 | 86 | 257 | 3 |
| *Plebeius argus* | 1 | 0 | 0 | 0 | 0 | 0 | 0 | 0 | 0 | 0 | 1 |
| *Polyommatus amandus* | 253 | 79 | 5 | 15 | 3 | 17 | 25 | 38 | 140 | 243 | 3 |
| *Nymphalis c–album* | 8 | 0 | 0 | 0 | 1 | 0 | 0 | 0 | 0 | 1 | 2 |
| *Polyommatus icarus* | 25 | 5 | 0 | 3 | 0 | 0 | 1 | 0 | 5 | 9 | 3 |
| *Polyommatus semiargus* | 72 | 15 | 0 | 0 | 0 | 0 | 1 | 3 | 19 | 23 | 3 |
| *Thymelicus lineola* | 97 | 15 | 11 | 5 | 261 | 117 | 167 | 185 | 12 | 758 | 3 |
| *Plebeius optilete* | 1 | 0 | 0 | 0 | 0 | 0 | 0 | 0 | 0 | 0 | 1 |
| *Vanessa atalanta* | 2 | 0 | 0 | 0 | 0 | 1 | 0 | 0 | 8 | 9 | 3 |
| **Noctuoid moths** |  |  |  |  |  |  |  |  |  |  |  |
| *Autographa gamma* | 5 | 0 | 11 | 7 | 7 | 34 | 1 | 7 | 12 | 79 | 3 |
| *Callistege mi* | 19 | 1 | 0 | 0 | 0 | 1 | 2 | 0 | 18 | 21 | 3 |
| *Colobochyla salicalis* | 2 | 0 | 0 | 0 | 0 | 0 | 0 | 0 | 0 | 0 | 1 |
| *Cryptocala chardinyi* | 16 | 1 | 0 | 0 | 67 | 17 | 46 | 51 | 2 | 183 | 3 |
| *Euclidia glyphica* | 594 | 40 | 2 | 48 | 68 | 162 | 188 | 185 | 695 | 1348 | 3 |
| *Polypogon tentacularius* | 37 | 0 | 0 | 0 | 0 | 2 | 53 | 16 | 22 | 93 | 3 |
| **Geometroid moths** |  |  |  |  |  |  |  |  |  |  |  |
| *Cabera pusaria* | 4 | 0 | 0 | 0 | 0 | 0 | 0 | 0 | 0 | 0 | 1 |
| *Camptogramma bilineatum* | 1 | 0 | 0 | 0 | 0 | 0 | 0 | 0 | 0 | 0 | 1 |
| *Chiasmia clathrata* | 930 | 41 | 0 | 8 | 3 | 7 | 90 | 159 | 1003 | 1270 | 3 |
| *Ematurga atomaria* | 192 | 8 | 0 | 0 | 2 | 2 | 27 | 7 | 43 | 81 | 3 |
| *Epirrhoe alternata* | 16 | 0 | 0 | 1 | 0 | 0 | 1 | 1 | 7 | 10 | 3 |
| *Eulithis populata* | 1 | 0 | 0 | 0 | 0 | 0 | 0 | 0 | 0 | 0 | 1 |
| *Eulithis pyraliata* | 4 | 0 | 0 | 0 | 0 | 0 | 0 | 0 | 0 | 0 | 1 |
| *Idaea pallidata* | 23 | 0 | 0 | 0 | 0 | 0 | 0 | 0 | 4 | 4 | 2 |
| *Idaea serpentata* | 38 | 0 | 0 | 0 | 0 | 1 | 5 | 0 | 7 | 13 | 3 |
| *Jodis putata* | 1 | 0 | 0 | 0 | 0 | 0 | 0 | 0 | 0 | 0 | 1 |
| *Lampropteryx suffumata* | 1 | 0 | 0 | 0 | 0 | 0 | 0 | 0 | 0 | 0 | 1 |
| *Lomographa bimaculata* | 4 | 0 | 0 | 0 | 0 | 0 | 0 | 0 | 0 | 0 | 1 |
| *Lomaspilis marginata* | 11 | 0 | 0 | 0 | 0 | 0 | 0 | 0 | 8 | 8 | 3 |
| *Odezia atrata* | 39 | 1 | 0 | 0 | 0 | 0 | 0 | 0 | 7 | 7 | 3 |
| *Petrophora chlorosata* | 2 | 0 | 0 | 0 | 0 | 0 | 0 | 0 | 0 | 0 | 1 |
| *Scotopteryx chenopodiata* | 348 | 11 | 2 | 46 | 371 | 245 | 723 | 584 | 16 | 1987 | 3 |
| *Scopula immorata* | 38 | 2 | 0 | 2 | 0 | 0 | 14 | 3 | 6 | 25 | 3 |
| *Scopula immutata* | 12 | 0 | 0 | 4 | 0 | 1 | 7 | 1 | 2 | 15 | 3 |
| *Siona lineata* | 13 | 2 | 0 | 2 | 4 | 5 | 5 | 1 | 2 | 19 | 3 |
| *Xanthorhoe ferrugata* | 1 | 0 | 0 | 0 | 0 | 0 | 0 | 0 | 0 | 0 | 1 |
| *Xanthorhoe montanata* | 11 | 0 | 0 | 1 | 3 | 27 | 9 | 5 | 4 | 49 | 3 |
| *Xanthorhoe spadicearia* | 4 | 0 | 0 | 0 | 0 | 0 | 0 | 0 | 0 | 0 | 1 |

* The records from summer 2011 are not directly comparable to earlier years because they are not based on standardized transect counts, but are mostly based on individuals that were captured in the release set-aside field and then marked and released in the mobility experiment. The records made in 2011 indicate that several new species (e.g. *Lycaena hippothoe* and *Odezia atrata*) had colonized the release set-aside field after the systematic monitoring ended in autumn 2008.

**Reference**

Alanen, E-L., Hyvönen, T., Lindgren, S., Härmä, O. & Kuussaari, M. (2011) Differential responses of bumblebees and diurnal Lepidoptera to vegetation succession in long-term set-aside. *Journal of Applied Ecology*, **48**, 1251–1259.

**Table S2.** Species traits of the studied butterfly species used in the mobility analyses. See Materials and methods for explanation of trait classes.

| **Species** | **Size** | **Hab_pref_** | **Hab_spec_** | **Hab_suit_** | **Host_pref_** | **Host_spec_** |
| --- | --- | --- | --- | --- | --- | --- |
| **Butterflies** |  |  |  |  |  |  |
| *Anthocharis cardamines* | 39,5 | Forest edge | Generalist | 1 | Other herbs | Polyphagous |
| *Aphantopus hyperantus* | 38,9 | Grassland | Generalist | 3 | Poaceae | Polyphagous |
| *Araschnia levana* | 35,1 | Forest edge | Specialist | 1 | Other herbs | Oligophagous |
| *Argynnis adippe* | 53,6 | Grassland | Specialist | 2 | Other herbs | Oligophagous |
| *Argynnis aglaja* | 53,1 | Grassland | Specialist | 2 | Other herbs | Oligophagous |
| *Aricia artaxerxes* | 28,1 | Grassland | Specialist | 2 | Other herbs | Oligophagous |
| *Boloria euphrosyne* | 37,3 | Forest edge | Generalist | 1 | Other herbs | Polyphagous |
| *Boloria selene* | 36,6 | Grassland | Specialist | 3 | Other herbs | Oligophagous |
| *Brenthis ino* | 37,7 | Grassland | Specialist | 3 | Other herbs | Polyphagous |
| *Callophrys rubi* | 23,9 | Forest edge | Specialist | 1 | Forest plants | Oligophagous |
| *Celastrina argiolus* | 26,3 | Forest edge | Specialist | 1 | Forest plants | Polyphagous |
| *Coenonympha glycerion* | 30,2 | Grassland | Specialist | 3 | Poaceae | Oligophagous |
| *Erebia ligea* | 41,4 | Forest edge | Specialist | 2 | Poaceae | Polyphagous |
| *Aricia eumedon* | 28,1 | Grassland | Specialist | 2 | Other herbs | Oligophagous |
| *Gonepteryx rhamni* | 49,8 | Forest edge | Generalist | 2 | Forest plants | Oligophagous |
| *Leptidea sinapis* | 38,3 | Forest edge | Specialist | 1 | Fabaceae | Polyphagous |
| *Lycaena hippothoe* | 31,3 | Grassland | Specialist | 3 | Other herbs | Oligophagous |
| *Lycaena virgaureae* | 25,9 | Grassland | Generalist | 2 | Other herbs | Oligophagous |
| *Melitaea athalia* | 35,3 | Forest edge | Specialist | 1 | Other herbs | Polyphagous |
| *Nymphalis antiopa* | 66,5 | Forest edge | Specialist | 1 | Forest plants | Polyphagous |
| *Nymphalis io* | 54,0 | Field margin | Generalist | 3 | Other herbs | Oligophagous |
| *Nymphalis urticae* | 47,3 | Field margin | Generalist | 3 | Other herbs | Oligophagous |
| *Ochlodes sylvanus* | 29,9 | Forest edge | Generalist | 3 | Poaceae | Polyphagous |
| *Pieris napi* | 40,2 | Field margin | Generalist | 3 | Other herbs | Polyphagous |
| *Plebeius argus* | 23,0 | Forest edge | Specialist | 1 | Fabaceae | Polyphagous |
| *Polyommatus amandus* | 31,4 | Grassland | Generalist | 3 | Fabaceae | Polyphagous |
| *Nymphalis c–album* | 45,8 | Forest edge | Generalist | 2 | Other herbs | Polyphagous |
| *Polyommatus icarus* | 28,5 | Grassland | Specialist | 3 | Fabaceae | Polyphagous |
| *Polyommatus semiargus* | 28,8 | Grassland | Specialist | 3 | Fabaceae | Polyphagous |
| *Thymelicus lineola* | 25,5 | Grassland | Generalist | 3 | Poaceae | Polyphagous |
| *Vanessa atalanta* | 59,2 | Field margin | Generalist | 3 | Other herbs | Polyphagous |
